# *In silico* drug screening by using genome-wide association study data repurposed dabrafenib, an anti-melanoma drug, for Parkinson’s disease

**DOI:** 10.1093/hmg/ddy279

**Published:** 2018-08-22

**Authors:** Takeshi Uenaka, Wataru Satake, Pei-Chieng Cha, Hideki Hayakawa, Kousuke Baba, Shiying Jiang, Kazuhiro Kobayashi, Motoi Kanagawa, Yukinori Okada, Hideki Mochizuki, Tatsushi Toda

**Affiliations:** 1 Division of Neurology/Molecular Brain Science, Kobe University Graduate School of Medicine, Kobe, Hyogo, Japan; 2 Department of Neurology, Osaka University Graduate School of Medicine, Suita, Osaka, Japan; 3 Department of Statistical Genetics, Osaka University Graduate School of Medicine, Suita, Osaka, Japan; 4 Laboratory of Statistical Immunology, Immunology Frontier Research Center (WPI-IFReC), Osaka University, Suita, Osaka, Japan; 5 Department of Neurology, Graduate School of Medicine, The University of Tokyo, Bunkyo, Tokyo, Japan

## Abstract

Parkinson’s disease (PD) is a neurodegenerative disorder characterized by dopaminergic neuron loss. At present, there are no drugs that stop the progression of PD. As with other multifactorial genetic disorders, genome-wide association studies (GWASs) found multiple risk loci for PD, although their clinical significance remains uncertain. Here, we report the identification of candidate drugs for PD by a method using GWAS data and *in silico* databases. We identified 57 Food and Drug Administration-approved drug families as candidate neuroprotective drugs for PD. Among them, dabrafenib, which is known as a B-Raf kinase inhibitor and is approved for the treatment of malignant melanoma, showed remarkable cytoprotective effects in neurotoxin-treated SH-SY5Y cells and mice. Dabrafenib was found to inhibit apoptosis, and to enhance the phosphorylation of extracellular signal-regulated kinase (ERK), and inhibit the phosphorylation of c-Jun NH_2_-terminal kinase. Dabrafenib targets B-Raf, and we confirmed a protein–protein interaction between B-Raf and Rit2, which is coded by *RIT2*, a PD risk gene in Asians and Caucasians. In *RIT2*-knockout cells, the phosphorylation of ERK was reduced, and dabrafenib treatment improved the ERK phosphorylation. These data indicated that dabrafenib exerts protective effects against neurotoxicity associated with PD. By using animal model, we confirmed the effectiveness of this *in silico* screening method. Furthermore, our results suggest that this *in silico* drug screening system is useful in not only neurodegenerative diseases but also other common diseases such as diabetes mellitus and hypertension.

## Introduction

Parkinson’s disease (PD) is the most common neurodegenerative movement disorder, and is characterized by the loss of dopaminergic neurons in the substantia nigra and the formation of Lewy bodies that are primarily composed of aggregated α-synuclein in the neurons (1). Despite extensive investigation, there are currently no disease-modifying drugs available that can halt the progression of PD. The discovery of new drugs is an expensive and time-consuming process. It takes ∼15 years and >$1 billion to develop and bring a new drug to market (2). Furthermore, <5% of the new molecules that enter Phase 1 clinical trials are approved by the US Food and Drug Administration (FDA) (3). Under such circumstances, drug repurposing, which is the identification of new indications for existing drugs, is thought to be a promising strategy for intractable diseases such as PD.

Genome-wide association study (GWAS) results have now been reported for many common adult diseases (metabolic, auto-immune and psychiatric and so on). The common form of PD is also a multifactorial disorder, and previous GWASs have identified several genetic loci as genetic risks for sporadic PD (4,5). In 2014, 24 risk loci for sporadic PD were reported from a meta-analysis of Caucasian GWASs (6). Although GWAS data have provided valuable biological insight into the molecular mechanisms of PD, translation of the genetic findings from GWAS into the clinic has remained limited. Recently, a new method of drug discovery utilizing risk genes from GWAS and computational databases were developed for rheumatoid arthritis (7). This *in silico* screening method was subsequently used to search for drugs for colorectal cancer and type 2 diabetes, and some drugs that have been approved for other diseases were identified as candidate drugs (8,9), although their biological effects *in vitro* or *in vivo* were uncertain.

In the present study, we applied this method to search for disease-modifying drugs for sporadic PD, and identified some candidate drugs. Then, we analyzed their neuroprotective effects in *in vitro* and *in vivo* PD models, and demonstrated that dabrafenib is a promising neuroprotective drug for PD.

## Results

### *In silico* identification of potential disease-modifying drugs

**Figure 1:**
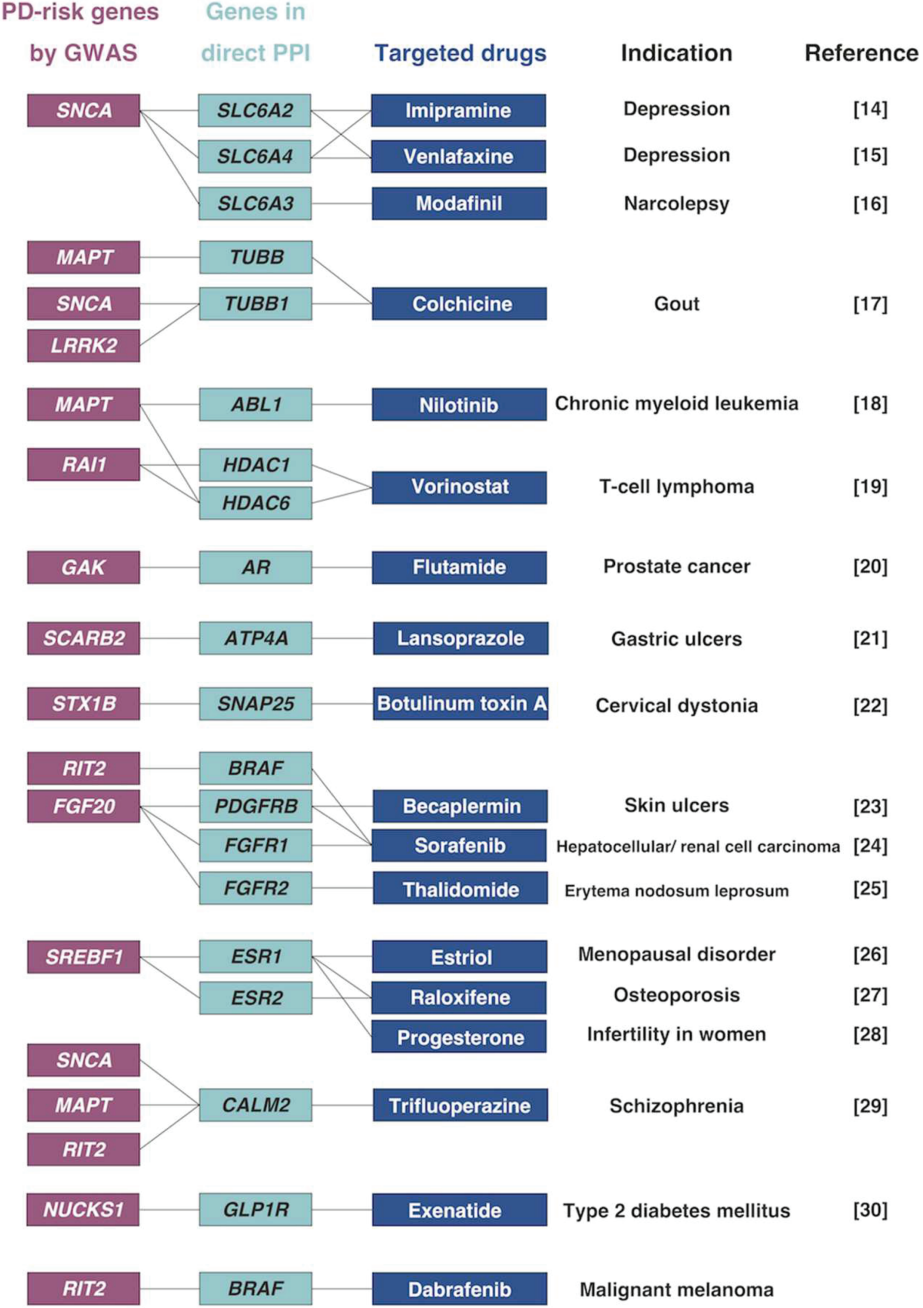
Examples of candidate drugs for PD identified by *in silico* drug screening ‘PD-risk genes’ were listed from the data of meta-GWAS for PD, and ‘genes in direct PPI’ were extracted using PPI databases. Using drug databases, we identified FDA-approved drugs that targeted ‘PD-risk genes’ or ‘genes in direct PPI’. These drugs are all approved for other diseases, and have already been reported as being neuroprotective for PD. We found dabrafenib as a new candidate drug for PD.

We applied the *in silico* screening method (7) to identify disease-modifying drugs for PD. We first defined 32 PD risk-genes within PD-risk loci that were detected in the previous meta-GWAS (6). Using protein–protein interaction (PPI) databases, InWeb (10) and PINA (11), we obtained 834 protein products showing direct PPI with protein products of the PD-risk genes. We considered that a total of 866 protein products from the 32 PD-risk genes and 834 genes in direct PPI have the possibility of involvement in PD pathogenesis. We further identified 871 drug target genes from the drug databases DrugBank (12) and Therapeutic Target Database (13). Among the 866 PD-risk/direct PPI genes, we found that 48 genes were targeted by 57 FDA-approved drug families for other diseases, and considered these to be candidate disease-modifying drugs for PD (Supplementary Material, Fig. S1). Neuroprotective effects in *in vitro* or *in vivo* PD model have already been reported in 17 of the 57 FDA-approved drug families (∼30%) (14–30) (Fig. 1). Therefore, our results suggest that this combinational analysis of GWAS-data and *in silico* database can efficiently identify drugs with neuroprotective effects.

### Neuroprotective effects of dabrafenib *in vitro* and *in vivo*

We then searched the previous literature for information on these candidates, focusing on the following five drugs that were expected to have neuroprotective effects from the data of other reports: dasatinib, duloxetine, furosemide, regorafenib and dabrafenib. Dasatinib was reported to show neuroprotective effects in *in vitro* and *in vivo* amyotrophic lateral sclerosis model (31). Duloxetine inhibited oxidative stress and apoptosis in rat pheochromocytoma-derived PC12 cells (32). Furosemide was reported to rescue the inhibitory effects of midazolam (benzodiazepine) on neural stem cell proliferation (33). Regorafenib was synthesized as a more potent multi-kinase inhibitor than sorafenib, which has already been reported to be neuroprotective in PD (24,34). Dabrafenib targets B-Raf, which was reported to be associated with neuronal survival (35). To examine whether these candidate drugs actually exert neuroprotective effects, we treated human neuroblastoma SH-SY5Y cells with each candidate drug together with 1-methyl-4-phenylpyridinium ion (MPP^+^), and performed the lactate dehydrogenase (LDH) assay and cell viability assay. MPP^+^ is a toxic molecule that is known to increase LDH and decrease cell viability as a result of mitochondrial dysfunction and apoptosis, and is hence thought to mimic the pathogenesis of PD (36).

**Figure 2:**
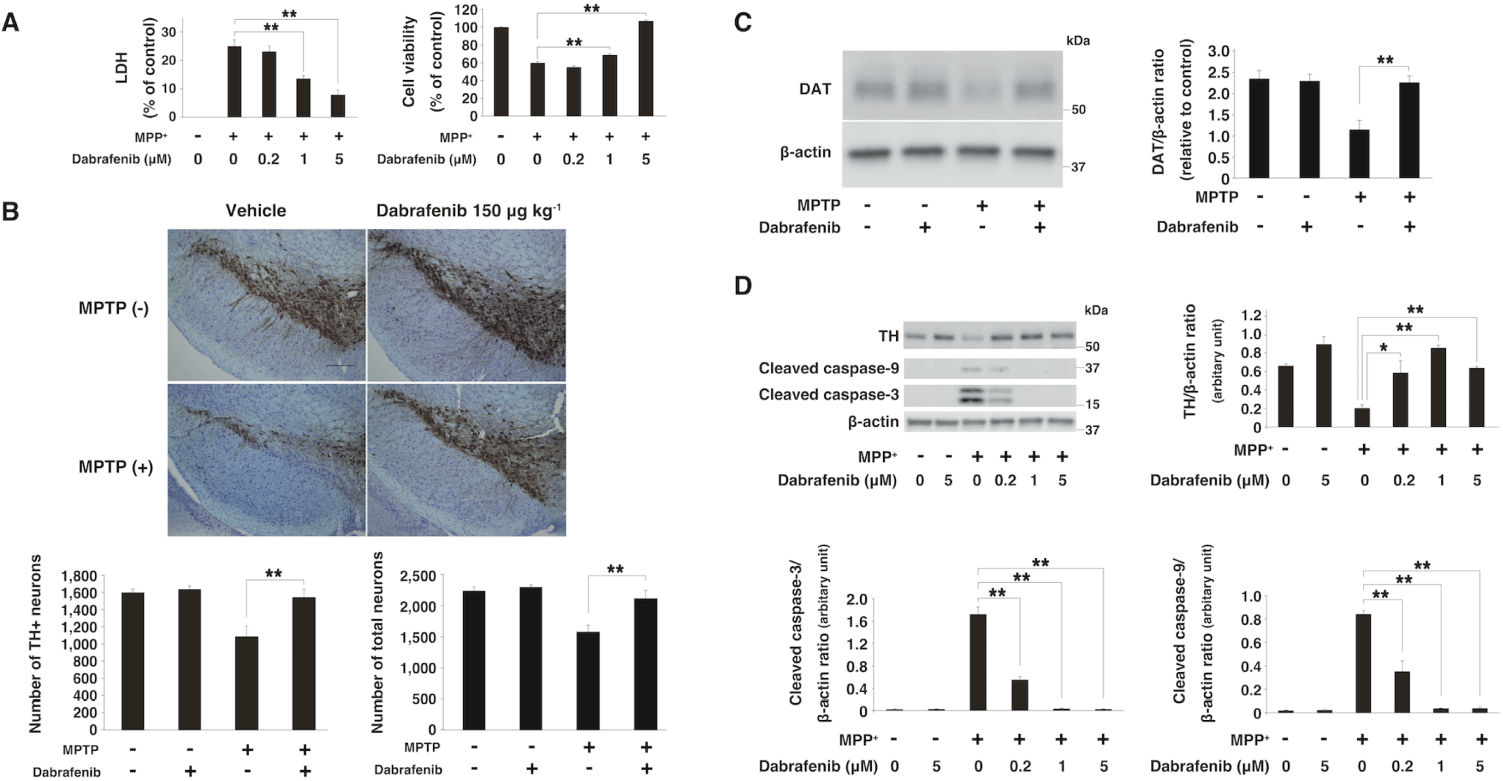
Neuroprotective effects of dabrafenib both *in vitro* and *in vivo* (**A**) LDH/cell viability assay under the condition of MPP^+^. Dabrafenib inhibited LDH release and the loss of cell viability at 24 h after MPP^+^ (3 mM) treatment in a concentration-dependent manner. Data represent the mean ± s.e.m. **, *P*-value < 0.01 compared with MPP^+^ alone (one-way ANOVA with the Tukey post-hoc test; n = 4 per group). (**B**) Immunohistochemistry staining of TH. Dabrafenib (150 μg kg^−1^) prevented the MPTP-induced loss of TH-positive and total neurons in the substantia nigra at 3 weeks after MPTP injection. Scale bar represents 200 μm. Data represent the mean ± s.e.m. ^**^, *P*-value < 0.01 compared with vehicle + MPTP group (one-way ANOVA with the Tukey post-hoc test; n = 5–9 per group). (**C**) Immunoblotting showed that dabrafenib rescued the loss of DAT in the substantia nigra of mice 3 weeks after MPTP injection. Data represent the mean ± s.e.m. ^**^, *P*-value < 0.01 compared with vehicle + MPTP group (one-way ANOVA with the Tukey post-hoc test; n = 3–4 per group). (**D**) Western blot analysis showed that dabrafenib reduced the levels of cleaved caspase-3 and 9 at 48 h after MPP^+^ (3 mM) treatment. Data are represented as the mean ± s.e.m. ^**^, *P*-value < 0.01 compared with MPP^+^ alone (one-way ANOVA with the Tukey post-hoc test; n = 3 per group).

Among these five drugs, we found that dabrafenib, a drug approved for melanoma, most robustly decreased LDH release (*P*-value < 0.01) and increased cell viability (*P*-value < 0.01) in cells exposed to MPP^+^ (Fig. 2A and Supplementary Material, Fig. S2).

To assess the effects of these drugs *in vivo*, we analyzed their neuroprotective effects in a subacute 1-methyl-4-phenyl-1, 2, 3, 6-tetrahydropyridine (MPTP) mice model. MPTP is a neurotoxin, which is known to be converted to MPP^+^ and reproduce most of the biochemical and pathological hallmarks of PD, including the extensive degeneration of dopaminergic neurons (36).

Intracerebroventricular (icv) injection of dabrafenib 3 days before MPTP administration significantly prevented the loss of tyrosine hydroxylase (TH)-positive and total neurons (*P*-value < 0.01) at 3 weeks after MPTP injection (Fig. 2B). We also investigated the expression of dopamine transporter (DAT), which mediates the active reuptake of dopamine from the synapse and is a principal regulator of dopaminergic neurotransmission. At 3 weeks after the administration of MPTP, the DAT expression was also rescued by dabrafenib (Fig. 2C). Icv injection of dabrafenib 3 days before MPTP administration also significantly inhibited TH-positive neuronal death in a dose-dependent manner, assessed 1 week after MPTP injection (Supplementary Material, Fig. S3A and B). These data further confirmed the neuroprotective effects of dabrafenib in an *in vivo* PD model.

**Figure 3:**
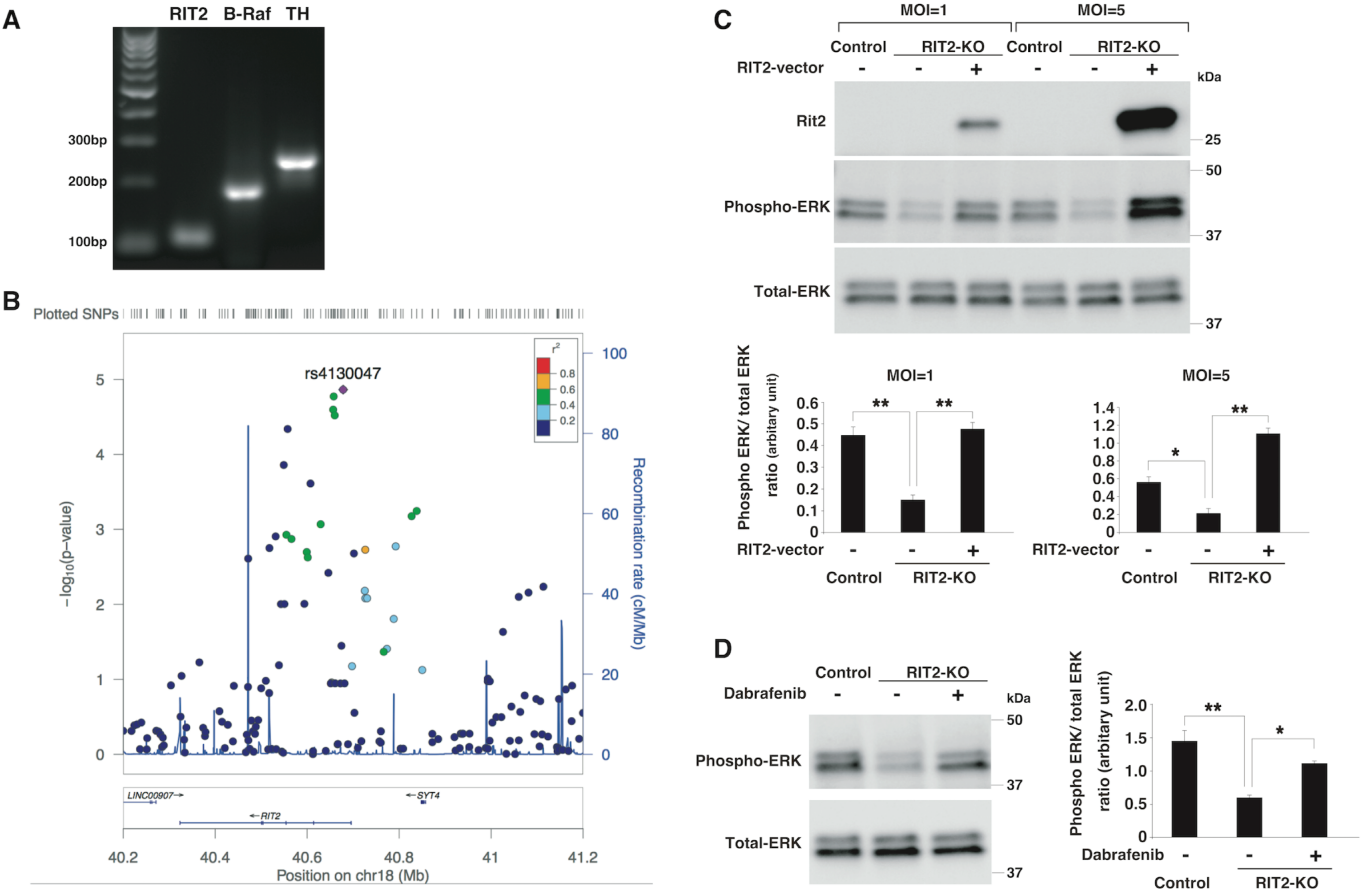
Significant association between *RIT2* and PD, and the effect of dabrafenib on *RIT2*-knockout (KO) cells (**A**) RT-PCR data for the *RIT2*, *BRAF* and *TH* expression in the mouse substantia nigra. (**B**) Regional association plot of the *RIT2* locus. (**C**) Immunoblot of phosphorylated ERK and Rit2. Phospho-ERK was reduced in *RIT2*-KO (clone 42) compared to control cells (clone 7), and overexpression of *RIT2* in *RIT2*-KO cells rescued the phosphorylation of ERK. (Upper) representative immunoblots and (bottom) summarized data of phospho-ERK/total ERK levels in each cell line. (**D**) Dabrafenib upregulated the expression of phospho-ERK in *RIT2*-KO cells (clone 42). (Left) representative immunoblots and (right) summarized data of phospho-ERK/total ERK levels in each cell line. Data in (C) and (D) represent the mean ± s.e.m. (^*^, *P*-value < 0.05 and ^**^, *P*-value < 0.01; one-way ANOVA with the Tukey post-hoc test; n = 3 per group).

Next, we analyzed the anti-apoptotic effects of dabrafenib, because vemurafenib, a selective inhibitor of B-Raf containing the melanoma-causing V600E pathogenic mutation (*BRAF*^V600E^), was reported to suppress apoptosis in non-*BRAF*^V600E^-mutated cells (37). Dabrafenib significantly reduced the expression of cleaved caspase-3 and caspase-9 under MPP^+^-treated conditions (Fig. 2D). Furthermore, dabrafenib rescued the expression of TH in the presence of MPP^+^ (Fig. 2D). Dabrafenib also tended to reduce the expression of cleaved caspase-3, induced by MPTP *in vivo*, although the effect was not significant (Supplementary Material, Fig. S3C). These results indicated the anti-apoptotic effects of dabrafenib. In this subacute MPTP mice model, dabrafenib did not affect the expression of other proteins such as α-synuclein, p62, glial fibrillary acidic protein (GFAP) and ionized calcium-binding adapter molecule 1 (IBA-1), which are known to be associated with the pathophysiology of PD (Supplementary Material, Fig. S3C).

### Expression of *BRAF* and *RIT2* and interaction of their encoded proteins

Dabrafenib targets B-Raf, which directly interacts with the protein product of the Ras-like without CAAX 2 (*RIT2*) gene. A previous report (38) and our co-immunoprecipitation experiment confirmed the interaction between Rit2 and B-Raf (Supplementary Material, Fig. S4A). We confirmed the expression of *RIT2* and *BRAF* transcripts in the mouse substantia nigra pars compacta (SNpc) by reverse transcription–polymerase chain reaction (RT-PCR) (Fig. 3A). The expression and phosphorylation of B-Raf were also observed in other brain regions, such as the cortex, hippocampus, striatum and cerebellum (Supplementary Material, Fig. S4B). The phosphorylation of B-Raf was not affected by MPTP or dabrafenib at 3 weeks after treatment (Supplementary Material, Fig. S4C). It has been reported that *RIT2* mRNA was specifically expressed in TH-positive neurons (39) and that *BRAF* mRNA was expressed in several brain regions, including SNpc, as shown by *in situ* hybridization (40), which is consistent with our results.

### PD-susceptibility of East Asians and Caucasians with *RIT2*

A significant association between PD and *RIT2* was reported in two meta-GWASs of Caucasian samples (6,41), but it remains unclear as to whether there is an association between PD and *RIT2* in other racial populations (42,43). To assess the association between *RIT2* and PD in the Asian population, we investigated the association between PD and single nucleotide polymorphisms (SNPs) within this locus identified from our GWAS data, which we previously conducted using genotype data from 435 470 SNPs in 988 PD cases and 2521 controls of Japanese origin (4). We found a positive association between *RIT2* and PD (rs4130047, P trend = 1.37 × 10^−5^ by the Cochran–Armitage trend test and 6.8 × 10^−5^ by a 2 × 3 chi-squared test) in the East Asian population (Fig. 3B and Table 1). Furthermore, we identified rs4130047 as the most significant SNP in this locus. This SNP is located within intron 1 of *RIT2*, and is located 4.8 kb away from rs12456492, the peak SNP reported in the Caucasian meta-GWASs (6,41). We did not have direct genotype data of rs12456492, because our genotyping array did not include this SNP, but HaploReg (44) indicated that rs4130047 is in perfect linkage disequilibrium with rs12456492 (r^2^ = 1) (Table 1). The detection of a positive association between *RIT2* and PD in the East Asian, as well as Caucasian population, demonstrates the definite PD susceptibility of this locus. We also performed a meta-analysis for the SNP rs4130047 using the MyMeta function implemented in the PDGene database (http://www.pdgene.org/my_meta). A forest plot indicated that in our study (4), SNP rs4130047 had an odds ratio of 1.27 (C versus T; 95% confidence interval = 1.14 – 1.41), and the association was in the same direction as in the original study (Supplementary Material, Table S1).

Although we also explored two eQTL databases, namely, the GTEx database (https://www.gtexportal.org/) and The Brain eQTL Almanac (www.Braineac.org), to examine potential influence of SNPs on the expression of *RIT2*, no convincing conclusion could be reached because of inconsistent reports on our target SNPs in the two databases.

### Rescue of the effects of *RIT2*-KO by dabrafenib

A reduced expression of *RIT2* was reported in the substantia nigra of patients with PD (45). To determine the effect of *RIT2* deficiency in neuronal cells, we created *RIT2*-KO SH-SY5Y cells by using CRISPR/Cas9 genome editing (Supplementary Material, Fig. S5A). *RIT2* is known to increase the expression of phosphorylated extracellular signal-regulated kinase (ERK) by activating B-Raf (38), and consistently, we observed a reduced expression of phospho-ERK in our *RIT2*-KO cells (Fig. 3C and Supplementary Material, Fig. S5B).

In *RIT2*-KO cells, rescue experiments with lentiviral overexpression of *RIT2* upregulated the phosphorylation of ERK in an expression level-dependent manner (Fig. 3C and Supplementary Material, Fig. S5B). These data confirmed that the reduction in phospho-ERK levels was caused by a loss of *RIT2*.

Previous studies demonstrated that adenosine 5′-triphospate (ATP)-competitive RAF inhibitors, such as dabrafenib, inhibit ERK signaling in cells with *BRAF*^V600E^, but enhance ERK signaling in cells with wild-type *BRAF* (37,46). Furthermore, we confirmed ERK activation by dabrafenib in SH-SY5Y cells (Supplementary Material, Fig. S6A). We found that treatment with dabrafenib improved the phosphorylation level of ERK in *RIT2*-KO cells (Fig. 3D and Supplementary Material, Fig. S5C).

### Inhibition of phosphorylated JNK/c-jun by dabrafenib

**Figure 4:**
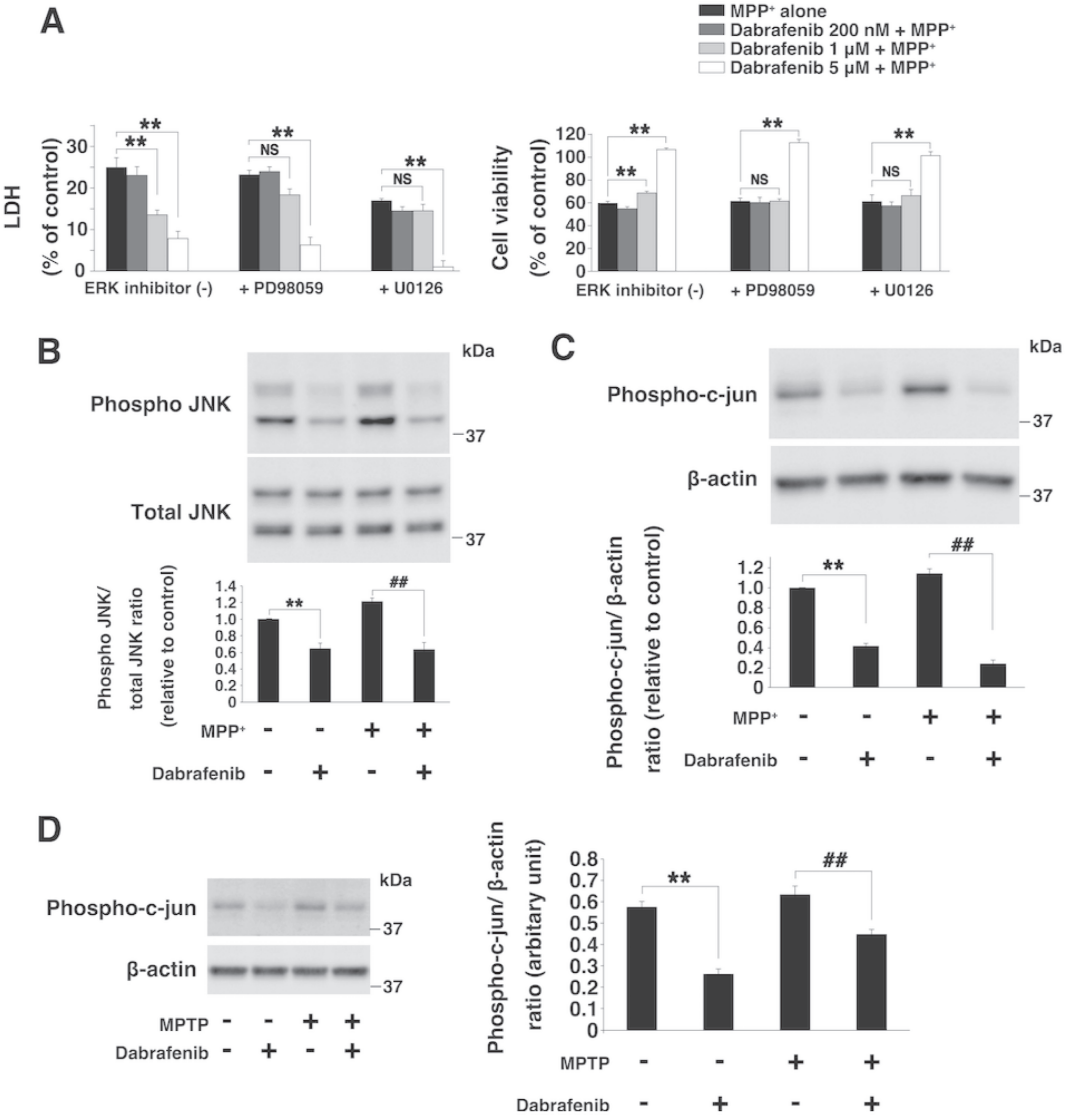
Dabrafenib reduced the expression of phosphorylated JNK and c-jun in both SH-SY5Y cells and mice (**A**) LDH/cell viability assay under the condition of ERK inhibitor. Neuroprotective effects of dabrafenib (5 μM) were not dependent on the downregulation of activated ERK expression by PD98059 (30 μM) or U0126 (3 μM). Neuroprotective effects were assessed by the LDH and cell viability assay in SH-SY5Y cells at 24 h after MPP^+^ (3 mM) exposure. ^**^, *P*-value < 0.01 compared with the MPP^+^ alone group; NS, not significant (n = 3 per group). (**B**) Immunoblot of phosphorylated JNK in SH-SY5Y cells at 30 min after drug treatment. Dabrafenib (5 μM) inhibited the phosphorylation of JNK with or without MPP^+^ (3 mM). ^**^, *P*-value < 0.01 compared with control; ##, *P*-value < 0.01 compared with MPP^+^ alone (n = 3 per group). (**C**) Immunoblot for phospho-c-jun in SH-SY5Y cells at 24 h after drug treatment. Dabrafenib (5 μM) decreased the expression of phospho-c-jun with or without MPP^+^ 3 mM. ^**^, *P*-value < 0.01 compared with control; ##, *P*-value < 0.01 compared with MPP^+^ alone (n = 3 per group). (**D**) Protein expression of phospho-c-jun in the substantia nigra of mice at 1 week after MPTP-injection. Dabrafenib significantly decreased the expression of phospho-c-jun with or without MPTP. ^**^, *P*-value < 0.01 compared with the normal control group; ##, *P*-value < 0.01 compared with the vehicle + MPTP group (n = 4–5 per group). Data represent the mean ± s.e.m. All statistical analyses in this figure were performed using one-way ANOVA with the Tukey post-hoc test.

To examine whether the neuroprotective effects of dabrafenib result from the activation of ERK, we inhibited ERK activity in cells using the ERK inhibitors PD98059 or U0126, and performed the LDH and cell viability assay. We found that the neuroprotective effects of dabrafenib (1 μM) were cancelled by ERK inhibition. On the other hand, these inhibitors could not block the neuroprotective effects of dabrafenib (5 μM), although ERK inhibitors suppressed ERK phosphorylation (Fig. 4A and Supplementary Material, Fig. S6B).

**Table 1:** Summary of association results for representative SNPs within the *RIT2* locus

SNP	hg19		Allele		MAF		Genotype count		P*_trend_*	SNP call	SNP call	P*_HWD_*	*r* ^2^ with
	Chr	position	Minor	Major	Case	Control	Case	Control		Case	Control	Control	rs12456492
rs879215	18	40557661	T	C	0.124	0.1626	19/207/762	58/704/1759	4.59 × 10^−5^	1.0000	1.0000	0.2146	< 0.2
rs4243267	18	40656531	G	T	0.3163	0.3697	99/427/462	340/1184/997	2.53 × 10^−5^	1.0000	1.0000	0.7324	0.42
rs4441358	18	40657643	G	A	0.3148	0.3693	97/428/463	340/1182/999	1.69 × 10^−5^	1.0000	1.0000	0.7648	0.44
rs4536548	18	40659924	A	G	0.3168	0.3697	99/428/461	340/1184/997	3.01 × 10^−5^	1.0000	1.0000	0.7324	0.42
rs4130047	18	40678235	C	T	0.4195	0.3635	176/477/335	330/1172/1018	1.37 × 10^−5^	1.0000	0.9996	0.8296	1

Nucleotide positions refer to hg19. P*_trend_* values were obtained using the Cochran–Armitage trend test (1 d.f.). MAF, minor allele frequency. P*_HWD_*, *P*-value of deviation from the Hardy–Weinberg equilibrium

These results indicated that although the neuroprotective effects of 1 μM of dabrafenib were dependent on ERK activation, the neuroprotective effects of 5 μM of dabrafenib were not completely dependent on ERK activation. Then, we investigated whether the c-Jun NH_2_-terminal kinase (JNK)/c-jun cascade was involved in the neuroprotective effects of dabrafenib, because a previous study reported that vemurafenib, a *BRAF*^V600E^ inhibitor similar to dabrafenib, also inhibited the phosphorylation of JNK (37). Furthermore, we found that dabrafenib downregulated the activation of JNK and c-jun independently of MPP^+^ exposure (Fig. 4B and C). These effects continued for at least 24 h (Supplementary Material, Fig. S6C). In MPTP-treated mice, dabrafenib also significantly decreased the expression of phospho-c-jun at 1 week after MPTP injection (Fig. 4D). These data suggested that dabrafenib demonstrated neuroprotective effects through the inhibition of JNK/c-jun, as well as the activation of ERK.

## Discussion

By utilizing GWAS data and *in silico* databases, we identified dabrafenib as a neuroprotective drug for sporadic PD, demonstrating the usefulness of this screening method practically. Dabrafenib exerted anti-apoptotic effects, activated ERK and inhibited JNK/c-jun phosphorylation. We showed the PD susceptibility of the *RIT2* gene in the Asian population, in addition to Caucasians, and showed that dabrafenib rescued the effects of *RIT2*-KO cells.

Dabrafenib (Tafinlar^Ⓡ^) was approved by the FDA in 2013 for the treatment of *BRAF*^V600E^ mutation-positive melanoma. Dabrafenib is known as a selective B-Raf kinase inhibitor (47), and induces cytotoxic effects specifically against cells with the *BRAF*^V600E^ mutation, which is observed in 40% of all melanoma patients (48). On the other hand, dabrafenib is reported to activate B-Raf in wild-type cells that do not have the *BRAF*^V600E^ mutation. B-Raf is a member of the Raf family, which comprises A-Raf, B-Raf and C-Raf. Cells of the central nervous system mainly express B-Raf and C-Raf, and the cytoplasmic concentration of B-Raf in neuronal cells appears higher than that of C-Raf (49). Previous reports demonstrated that B-Raf plays a role in neuronal survival (35) and maturation (50). These data indicated the close association between B-Raf and the nervous system.

Our data confirmed that dabrafenib activated ERK in SH-SY5Y cells, and the cytoprotective effects of dabrafenib (1 μM) were dependent on ERK activation (Fig. 4A). It was reported that activation of ERK showed neuroprotective effects in a PD model (14,51). We also investigated the effect of *RIT2* deficiency in SH-SY5Y cells because the expression of *RIT2* was decreased in the substantia nigra of PD patients. In *RIT2*-KO cells, phosphorylated ERK was suppressed, and this suppression was ameliorated by dabrafenib treatment. Other candidate of neuroprotective drug for PD, nilotinib (Abl-inhibitor), was identified on the basis of the fact that Abl levels were increased in the nigrostriatal region of PD patients (18). Our data demonstrated that dabrafenib improved the phosphorylation level of ERK in *RIT2*-KO cells. The viability of *RIT2*-KO cells was lower than that of control cells, but lentiviral overexpression of *RIT2* (multiplicity of infection of 10) increased the cell viability (Supplementary Material, Fig. S5D). However, treatment of *RIT2*-KO cells with dabrafenib failed to improve the cell viability (Supplementary Material, Fig. S5E). It is possible that the influence of the complete deletion of *RIT2* was severe and thus negated the effect of dabrafenib on cell viability. It is also possible that the complete deletion of *RIT2* may have changed several cellular pathways, thus suppressing the improvement in cell viability.

On the other hand, our data indicated that the neuroprotective effects of dabrafenib (5 μM) were not completely dependent on ERK activation. Dabrafenib at this concentration also inhibited JNK/c-jun phosphorylation. Some reports suggested that the inhibition of JNK had neuroprotective effects in both *in vitro* and *in vivo* PD models (52). Thus, we concluded that dabrafenib (5 μM) showed neuroprotective effects by the inactivation of JNK/c-jun, in addition to the activation of ERK.

We further found that dabrafenib exerted anti-apoptotic effects. Apoptosis is known to be one of the mechanisms contributing to the etiology of PD (53). An association between the overexpression of B-Raf and anti-apoptotic effects was reported (54). Furthermore, the activation of ERK (55) and the suppression of JNK (55) were found to inhibit apoptosis. These data suggested the possibility that dabrafenib exerted anti-apoptotic effects through the activation of ERK and the inactivation of JNK.

It is interesting that *in silico* drug screening using GWAS data of PD identified the anti-melanoma drug dabrafenib, because many epidemiological studies have demonstrated associations between PD and melanoma (56,57). Our present study is the first to demonstrate the neuroprotective effects of an anti-melanoma drug in a PD model. Peak serum concentrations of dabrafenib at the clinically used dose (150 mg twice daily) are equivalent to 1.55 μM *in vitro* (58), which is similar to the concentrations in our experiment.

Previous studies have reported that oral administration of dabrafenib could reduce the size of brain metastases in patients with malignant melanoma (58,59), which suggested a good penetration ability of dabrafenib to the central nervous system. It is also possible that dabrafenib may be injected to PD patients intrathecally, similar to the administration of nusinersen (Spinraza®) to patients with spinal muscular atrophy (60).

Several studies have suggested that L-3,4-dihydroxyphenylalanine (L-DOPA)-induced dyskinesia is associated with the activation of ERK (61). Dabrafenib also activates ERK in cells with wild-type *BRAF* and thus may potentially exacerbate dyskinesia. However, a previous report has demonstrated that the severity of L-DOPA-induced dyskinesia correlated with the extent of long-term ERK activation and that no persistent ERK activation was detected in the non-dyskinetic group (62). Our experiment with SH-SY5Y cells showed that the activation of ERK by dabrafenib was transient (< 24 h), suggesting a relatively low risk of dyskinesia (Supplementary Material, Fig. S6A). However, in real patients, the duration of ERK activation by dabrafenib will depend on the dosing regimen. Therefore, the best schedule of its administration should be considered to avoid persistent activation of ERK and to minimize the risk of dyskinesia.

Drug repurposing is a promising strategy for drug development because the safety data of these drugs in human patients are already available (63). However, it is inefficient to examine all FDA-approved drugs, because of their vast number. This *in silico* drug screening, which links the data of GWAS to drug/PPI databases, may narrow down candidate drugs for many polygenic diseases and save cost and time. Our present study is the first to demonstrate the efficacy of this drug screening method by using *in vitro* and *in vivo* PD model. In this study, we used the LDH/cell viability assay to verify the neuroprotective effects of candidate drugs. This enabled the identification of drugs with anti-apoptotic effects. Further studies are required to identify drugs with other neuroprotective effects, such as those that inhibit the aggregation or propagation of α-synuclein, by performing other assays. This screening method is also a promising strategy to identify fundamental therapeutic agents for not only other neurodegenerative diseases, such as Alzheimer’s disease and amyotrophic lateral sclerosis, but also many multifactorial genetic disorders, like diabetes mellitus and hypertension.

## Materials and Methods

### Materials

Dabrafenib was obtained from Cayman Chemical Company (Ann Arbor, MI, USA) and Selleck Chemicals (Houston, TX, USA). MPP^+^, MPTP-HCl and duloxetine were from Sigma-Aldrich (St. Louis, MO, USA). PD98059 and U0126 were from Cayman Chemical Company. Regorafenib was obtained from Adooq Bioscience (Irvine, CA, USA). Dasatinib was from BioVision (Milpitas, CA, USA). Furosemide was obtained from Abcam (Cambridge, MA, USA).

Antibodies against caspase-3 (#9665, 1:1000), cleaved caspase-9 (#7237, 1:1000), ERK 1/2 (#4695, 1:1000), phospho-ERK 1/2 (#4370, 1:2000), JNK (#9252, 1:1000), phospho-JNK (#4668, 1:1000), phospho-c-jun (#3270, 1:1000) and phospho-B-Raf (Ser445; #2696, 1:1000) were all purchased from Cell Signaling Technology (Beverly, MA, USA). The antibody against β-actin (A1978, 1:2000) was from Sigma-Aldrich. An anti-DAT antibody (MAB369, 1:1,000) was from Merck Millipore (Darmstadt, Germany). An anti-p62/SQSTM1 antibody (18420–1-AP, 1:1000) was from Proteintech (Chicago, IL, USA). An anti-α-synuclein antibody (610787, 1:1000) was from BD Transduction Laboratories (San Diego, CA, USA). An anti-IBA-1 antibody (016–20001, 1:1000) was from Wako (Osaka, Japan). An anti-GFAP antibody (Z0334, 1:2000) was from Dako (Copenhagen, Denmark). An anti-TH antibody (657012, 1:5000) was from Calbiochem (San Diego, CA, USA). Antibodies against Rit2 (sc-58474, 1:200), B-Raf (sc-5284, 1:200) and c-Myc (sc-40, 1:200) were from Santa Cruz Biotechnology (Santa Cruz, CA, USA). An anti-V5 antibody (04434–36, 1:1000) was from Nacalai Tesque (Kyoto, Japan).

### *In silico* drug screening

We focused on the candidate PD-risk genes that were identified by a meta-analysis of GWASs (6); *GBA, SYT11, RAB7L1, NUCKS1, SIPA1L2, ACMSD, TMEM163, STK39, MCCC1, TMEM175, GAK, DGKQ, BST1, FAM47E, SCARB2, SNCA, HLA-DQB1, GPNMB, INPP5F, MIR4697, LRRK2, CCDC62, GCH1, VPS13C, BCKDK, STX1B, MAPT, RIT2, DDRGK1, FGF20, SREBF1* and *RAI1*. We performed an interaction simulation of these PD-risk genes using InWeb (10) and PINA (11) databases, and identified genes in direct PPI with the PD-risk genes. PD-risk genes by GWAS and genes in direct PPI were combined as PD-associated genes. Next, we obtained all genes that were targeted by drugs from DrugBank (12) and Therapeutic Targets Database (13). We identified the overlapping genes between the PD-associated genes and all drug target genes. Drugs that targeted these overlapping genes were identified as candidate drugs for PD.

### Cell culture

Human neuroblastoma SH-SY5Y cells (a kind gift from Dr Miho Murata, National Center Hospital, National Center of Neurology and Psychiatry) and HEK293T cells were maintained in Dulbecco’s modified Eagle’s medium (DMEM) with 10% fetal bovine serum, 1% penicillin/streptomycin at 37°C in a humidified incubator with a 5% CO_2_ environment. All experiments were carried out 24–48 h after the cells were seeded.

### Measurement of LDH release and cell viability

SH-SY5Y cells (2 × 10^4^) were grown on collagen-coated 96-well plates in serum-free medium. Twenty-four h later, cells were treated with candidate drugs, followed by MPP^+^ (3 mM) for another 24 h. After incubation, the release of LDH was measured with a cytotoxicity detection kit (Roche Applied Science, Indianapolis, IN, USA). For measurement of cell viability, the CellTiter 96 AQueous One Solution cell proliferation assay (Promega, Madison, WI, USA) was used. We performed these assays according to the manufacturer’s instructions, and measured absorbance at 490 nm using a microplate reader (Molecular Devices, Sunnyvale, CA, USA). To compare cell viability between control and *RIT2*-KO cells, absorbance was measured 48 h after the administration of dabrafenib or lentiviral overexpression of *RIT2*.

### Animals

C57BL/6J mice (8–12 weeks old; 20–25 g; Charles River Laboratories, Kingston, NY, USA) were used. Animals were housed five per cage in a temperature-controlled room under a 12 h light/dark cycle with *ad libitum* access to food and water. All animal experiments were conducted according to the Osaka University Medical School Guidelines for the Care and Use of Laboratory Animals.

All animal procedures were approved by the Osaka University Medical School Animal Care and Use Committee.

### Drug treatment

Mice were anesthetized using sodium pentobarbital (Kyoritsu Seiyaku, Tokyo, Japan), injected intraperitoneally and placed in a stereotaxic instrument (Narishige, Tokyo, Japan). Using a 10 μL Hamilton syringe, vehicle (5% dimethylsulfoxide and polyethylene glycol 300) or dabrafenib (150 μg kg^−1^, 15 μg kg^−1^ or 1.5 μg kg^−1^ per mouse) was injected into the lateral ventricle of mice at the following coordinates: 0.2 mm posterior to bregma, 1 mm lateral to the midline and 3 mm ventral to the skull surface.

Three days after the treatment, the mice were intraperitoneally injected with MPTP-HCl (30 mg kg^−1^; Sigma) in saline daily for five consecutive days. The mice were subsequently sacrificed 7 days or 21 days after the last administration of MPTP.

### Immunohistochemistry

After anesthetization with sodium pentobarbital, mice were perfused transcardially with phosphate-buffered saline (PBS), followed by 4% paraformaldehyde in PBS. Then, the brains were removed and immersed for 48 h in 4% paraformaldehyde at 4°C and in 30% sucrose containing 0.01% sodium azide for another 48 h at 4°C. Nigral coronal sections (20 μm thick) were prepared with a cryostat. The sections were first rinsed with PBS, and then they were immersed in a solution of PBS containing 3% hydrogen peroxidase for 10 min. After washing with PBS, the sections were incubated overnight at 4°C with primary antibodies diluted with blocking solution (PBS-T containing 10% Block-Ace; DS Pharma Biomedical, Osaka, Japan). The sections were washed with PBS, and incubated with biotinylated secondary antibodies (VECTOR Laboratories, Burlingame, CA, USA, 1:500) in PBS-T for 1 h and then with VECTASTAIN ABC reagent (VECTOR Laboratories) for 1 h at room temperature. Finally, the sections were incubated for 5 min with 3,3′-diaminobenzidine (Sigma) for visualization.

### Stereological cell counting

The number of dopaminergic neurons in the SNpc was analyzed by unbiased stereological counting method (64). TH- and Nissl-double-positive neurons and total (i.e. Nissl-positive) neurons were counted in the right and left SNpc of every fourth 20 μm section throughout the entire extent of the SNpc. To avoid double counting of neurons with unusual shapes, TH- and Nissl-double-positive neurons were counted only when their nuclei and nucleoli were optimally visualized.

### Western blot analysis

SH-SY5Y cells were seeded at a density of 0.5–1 × 10^6^ cells in six-well plates and treated with dabrafenib and/or MPP^+^ for various times. Cells were lysed with RIPA buffer (50 mM Tris–HCl, 150 mM NaCl, 1% NP-40, 0.5% deoxycholate and 0.1% sodium dodecyl sulfate [SDS]), or SDS buffer (62.5 mM Tris–HCl, 2% SDS and 10% glycerol) and ultrasonicated for ∼20 s. The substantia nigra were isolated from each mouse brain, and then lysed in CelLytic MT Cell Lysis Reagent (#C3228, Sigma) or SDS buffer followed by ultrasonication. Protein concentrations were estimated using the BCA Protein Assay Kit (Thermo Fisher Scientific, Waltham, MA, USA). Proteins (10–20 μg) were separated by 4–15% SDS-polyacrylamide gradient gel electrophoresis (BioRad, Hercules, CA, USA), followed by transfer to a polyvinylidene difluoride membrane (Merck Millipore). Membranes were blocked with 5% skim milk in TBS-T for 1 h, and then incubated with a primary antibody overnight at 4°C. After incubation with an appropriate secondary antibody for 1 h at room temperature, protein bands were visualized by the ECL western blotting detection system (GE Healthcare, Madison, WI, USA). The bands were semi-quantified by densitometric analysis with Image J software.

### RT-PCR

Total RNA was isolated from the SNpc of a control mouse using TRIzol (Invitrogen, Carlsbad, CA, USA) and converted to cDNA using SuperScript III reverse transcriptase (Invitrogen) in accordance with the manufacturer’s protocols. The primers for mouse *RIT2* were 5′-GATGATGCTTTTCAAGGCTT-3′ and 5′-GGCTTTTATCTTCTTCCACA-3′; primers for mouse *BRAF* were 5′-GCTCTGTTCAATGGCGACATGG-3′ and 5′-AGGCCTCCAGGTATATTGATGGTG-3′ and primers for mouse *TH* were 5′-TACGCCACGCTGAAGGGCCTCTAT-3′ and 5′-AGGTGAGGAGGCATGACGGATGTA-3′.

### Association study and genomic analysis

We used summary statistics of the Cochran–Armitage trend test from our previous GWAS, which consisted of 435 470 SNPs in 988 PD and 2521 control Japanese samples (4). We generated regional plots presenting the *P*-values of SNPs across the *RIT2* locus (40.2–41.2 Mb on chromosome 18) using LocusZoom (65). We used linkage disequilibrium information from the Asian population of 1000 Genomes. We examined r^2^ values between rs12456492 and each SNP using HaploReg (Asian population of 1000 Genomes) (44). The MyMeta function implemented in the PDGene database (http://www.pdgene.org/my_meta) was used to perform a meta-analysis for the SNP rs4130047.

### Co-immunoprecipitation

Mammalian expression constructs containing the human c-Myc-tagged *RIT2* or V5-tagged *BRAF* in the pcDNA3.1/*myc*-His A vector (Invitrogen) were generated by PCR amplification. To examine the interaction between the Rit2 and B-Raf proteins, HEK293T cells were transiently transfected with the plasmids encoding c-Myc-Rit2 or V5-B-Raf using Effectene (Qiagen, Hilden, Germany). After 48 h, the cells were lysed with RIPA buffer containing protease and phosphatase inhibitors. The cell lysates were incubated with anti-c-Myc antibody-conjugated agarose beads (M047–8; MBL, Woburn, MA, USA) or anti-V5 antibody-conjugated agarose beads (PM003–8; MBL). After the incubation, the beads were washed five times and subjected to immunoblot analysis with the indicated antibodies.

### Preparation of *RIT2*-KO cells with CRISPR/Cas9 gene editing

We used the Cas9 Smart Nuclease All-in-One vector (System Biosciences, Mountain View, CA, USA). The CRISPR/Cas9 targeting sequence for *RIT2* was GATTATCATGACCCTACTAT. The All-in-One vector was transfected into SH-SY5Y cells together with the zeocin-resistant gene in pcDNA3.1/*myc*-His A (20:1 ratio) using Lipofectamine 2000 reagent (Thermo Fisher Scientific) according to the manufacturer’s protocol. The resulting zeocin-resistant colonies were dissociated into single cells and plated at 5000 cells per 10 cm dish. Mutations in the cell clones were verified by DNA sequencing of *RIT2*. The mutations in each clone were as follows: clone 13 (homozygous 1 bp insertion in exon 2); clone 21 (homozygous 1 bp deletion in exon 2) and clone 42 (homozygous 2 bp deletion in exon 2). We used clones that underwent genome editing but revealed no mutation in *RIT2* as controls (clones 7, 8 and 9).

### Lentiviral infection

The lentivirus vector CSII-CMV-MCS-IRES2-Venus, and the two packaging vectors pCAG-HIVgp and pCMV-VSV-G-RSV-Rev, were kindly provided by Dr Hiroyuki Miyoshi (Riken BioResource Center, Tsukuba, Japan). To generate the lentiviruses, the recombinant *RIT2* vector was transiently transfected into Lenti-X 293 T cells (Clontech, Mountain View, CA, USA) together with two packaging vectors. Forty-eight h after transfection, the viral supernatants were concentrated using a LentiX concentrator (Clontech). Viral particles were resuspended into DMEM. Analysis of lentiviral experiments was performed 2 days post infection at a multiplicity of infection of 1, 5 and 10.

### Statistical analysis

Data are expressed as the mean ± s.e.m. All data were acquired from at least three independent experiments. Statistical analyses were performed by one-way ANOVA followed by the Tukey’s post-hoc test. A *P*-value of >0.05 was considered to indicate a statistically significant difference between two groups.

## Supplementary Material

Supplementary Data
