# Supplementary material for: In silico drug screening by using genome-wide association study data repurposed dabrafenib, an anti-melanoma drug, for Parkinson’s disease
Source: Hum Mol Genet. 2018 Aug 22;27(22):3974–85. doi: 10.1093/hmg/ddy279 (PMC6216208; doi:10.1093/hmg/ddy279)
Supplement: Supplementary Data [file ddy279_supp.pdf]

Supplementary Material

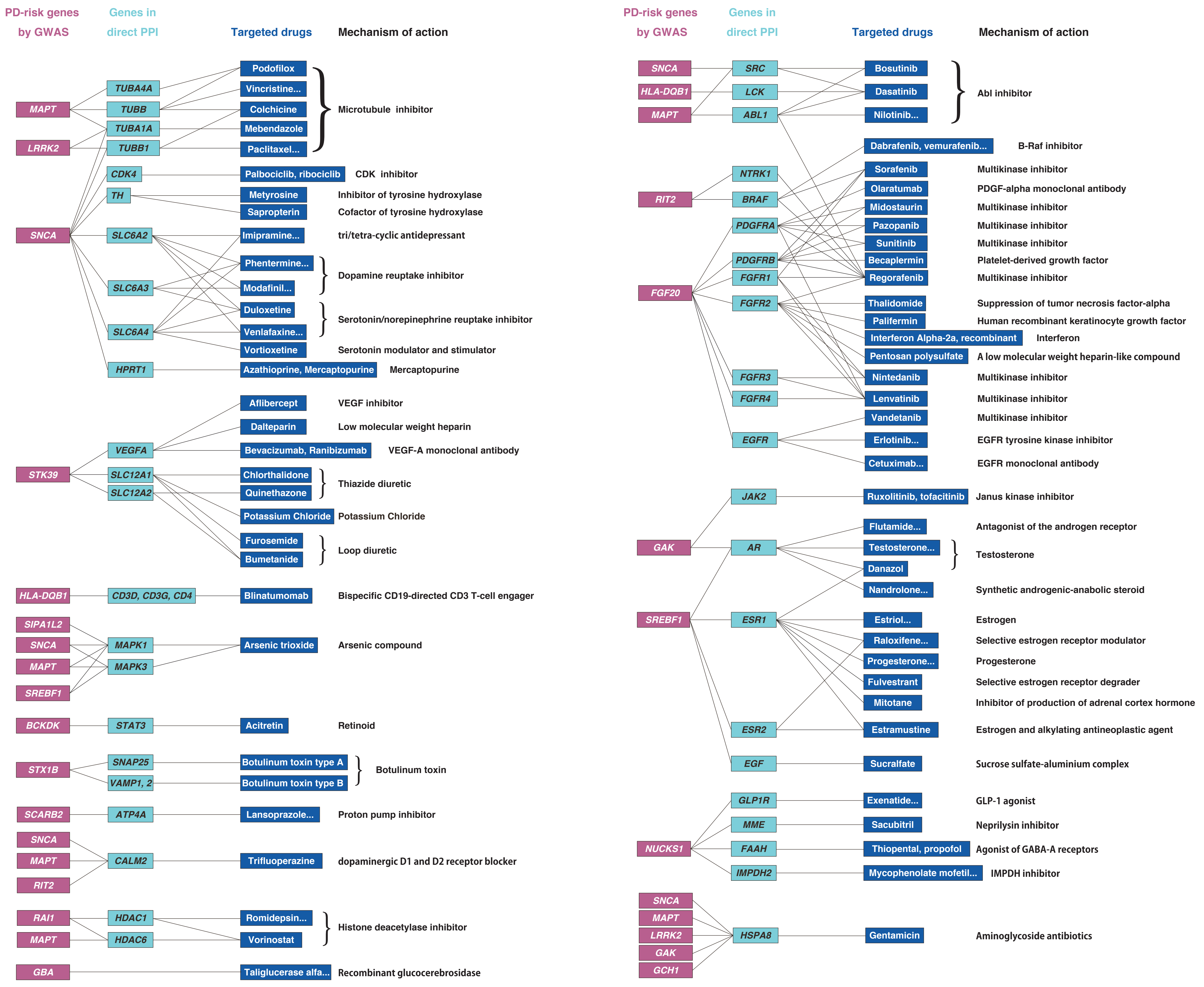
PD-risk genes by GWAS
Genes in direct PPI
Targeted drugs
Mechanism of action
MAPT
LRRK2
SNCA
TUBA4A
TUBB
TUBA1A
TUBB1
CDK4
TH
SLC6A2
SLC6A3
SLC6A4
HPRT1
Podofilox
Vincristine...
Colchicine
Mebendazole
Paclitaxel...
Microtubule inhibitor
Palbociclib, ribociclib
CDK inhibitor
Metyrosine
Inhibitor of tyrosine hydroxylase
Sapropterin
Cofactor of tyrosine hydroxylase
Imipramine...
tri/tetra-cyclic antidepressant
Phentermine...
Modafinil...
Dopamine reuptake inhibitor
Duloxetine
Venlafaxine...
Serotonin/norepinephrine reuptake inhibitor
Vortioxetine
Serotonin modulator and stimulator
Azathioprine, Mercaptopurine
Mercaptopurine
STK39
VEGFA
SLC12A1
SLC12A2
Aflibercept
VEGF inhibitor
Dalteparin
Low molecular weight heparin
Bevacizumab, Ranibizumab
VEGF-A monoclonal antibody
Chlorthalidone
Quinethazone
Thiazide diuretic
Potassium Chloride
Potassium Chloride
Furosemide
Bumetanide
Loop diuretic
HLA-DQB1
CD3D, CD3G, CD4
Blinatumomab
Bispecific CD19-directed CD3 T-cell engager
SIPA1L2
SNCA
MAPT
SREBF1
MAPK1
MAPK3
Arsenic trioxide
Arsenic compound
BCKDK
STAT3
Acitretin
Retinoid
STX1B
SNAP25
VAMP1, 2
Botulinum toxin type A
Botulinum toxin type B
Botulinum toxin
SCARB2
ATP4A
Lansoprazole...
Proton pump inhibitor
SNCA
MAPT
RIT2
CALM2
Trifluoperazine
dopaminergic D1 and D2 receptor blocker
RAI1
MAPT
HDAC1
HDAC6
Romidepsin...
Vorinostat
Histone deacetylase inhibitor
GBA
Taliglucerase alfa...
Recombinant glucocerebrosidase
PD-risk genes by GWAS
Genes in direct PPI
Targeted drugs
Mechanism of action
SNCA
HLA-DQB1
MAPT
SRC
LCK
ABL1
Bosutinib
Dasatinib
Nilotinib...
Abl inhibitor
Dabrafenib, vemurafenib...
B-Raf inhibitor
RIT2
FGF20
NTRK1
BRAF
PDGFRA
PDGFRB
FGFR1
FGFR2
FGFR3
FGFR4
EGFR
Sorafenib
Multikinase inhibitor
Olaratumab
PDGF-alpha monoclonal antibody
Midostaurin
Multikinase inhibitor
Pazopanib
Multikinase inhibitor
Sunitinib
Multikinase inhibitor
Becaplermin
Platelet-derived growth factor
Regorafenib
Multikinase inhibitor
Thalidomide
Suppression of tumor necrosis factor-alpha
Palifermin
Human recombinant keratinocyte growth factor
Interferon Alpha-2a, recombinant
Interferon
Pentosan polysulfate
A low molecular weight heparin-like compound
Nintedanib
Multikinase inhibitor
Lenvatinib
Multikinase inhibitor
Vandetanib
Multikinase inhibitor
Erlotinib...
EGFR tyrosine kinase inhibitor
Cetuximab...
EGFR monoclonal antibody
GAK
SREBF1
JAK2
AR
ESR1
ESR2
EGF
Ruxolitinib, tofacitinib
Janus kinase inhibitor
Flutamide...
Antagonist of the androgen receptor
Testosterone...
Danazol
Testosterone
Nandrolone...
Synthetic androgenic-anabolic steroid
Estriol...
Estrogen
Raloxifene...
Selective estrogen receptor modulator
Progesterone...
Progesterone
Fulvestrant
Selective estrogen receptor degrader
Mitotane
Inhibitor of production of adrenal cortex hormone
Estramustine
Estrogen and alkylating antineoplastic agent
Sucralfate
Sucrose sulfate-aluminium complex
NUCKS1
GLP1R
MME
FAAH
IMPDH2
Exenatide...
GLP-1 agonist
Sacubitril
Neprilysin inhibitor
Thiopental, propofol
Agonist of GABA-A receptors
Mycophenolate mofetil...
IMPDH inhibitor
SNCA
MAPT
LRRK2
GAK
GCH1
HSPA8
Gentamicin
Aminoglycoside antibiotics

Supplementary Figure S1. Connection between PD risk genes and FDA-approved drugs.

Full lists of the connections between PD risk genes by GWAS (magenta boxes), genes from the expanded PPI network (turquoise boxes), and FDA-approved drugs (blue boxes). Drugs were divided into 57 families by their mechanisms of action. Black lines indicate connections.

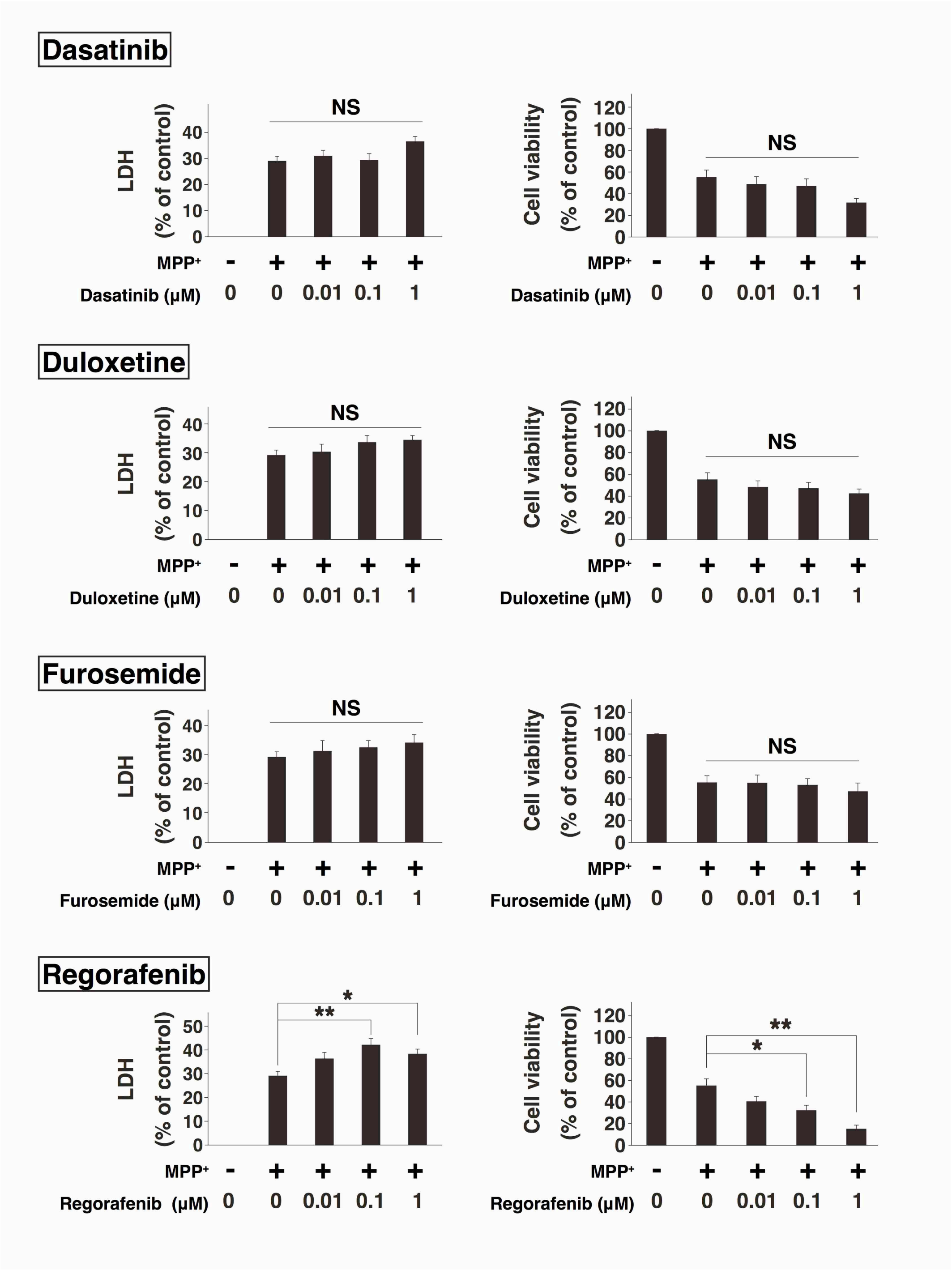

Supplementary Figure S2. Results of the LDH and cell viability assay of cells treated with the candidate drugs.

Cells were treated with the indicated drugs, and LDH release and cell viability was measured 24 hours after MPP^+ (3 mM) treatment. Dasatinib, duloxetine, furosemide, and regorafenib could not inhibit LDH release and the loss of cell viability at 24 hours after MPP^+ (3 mM) treatment. Regorafenib also showed

significant cytotoxic effects. Data represent the mean ± s.e.m. *, $p < 0.05$ and **, $p < 0.01$ compared with MPP^+ alone; NS, not significant (one-way ANOVA with the Tukey post-hoc test; n = 3 per group).

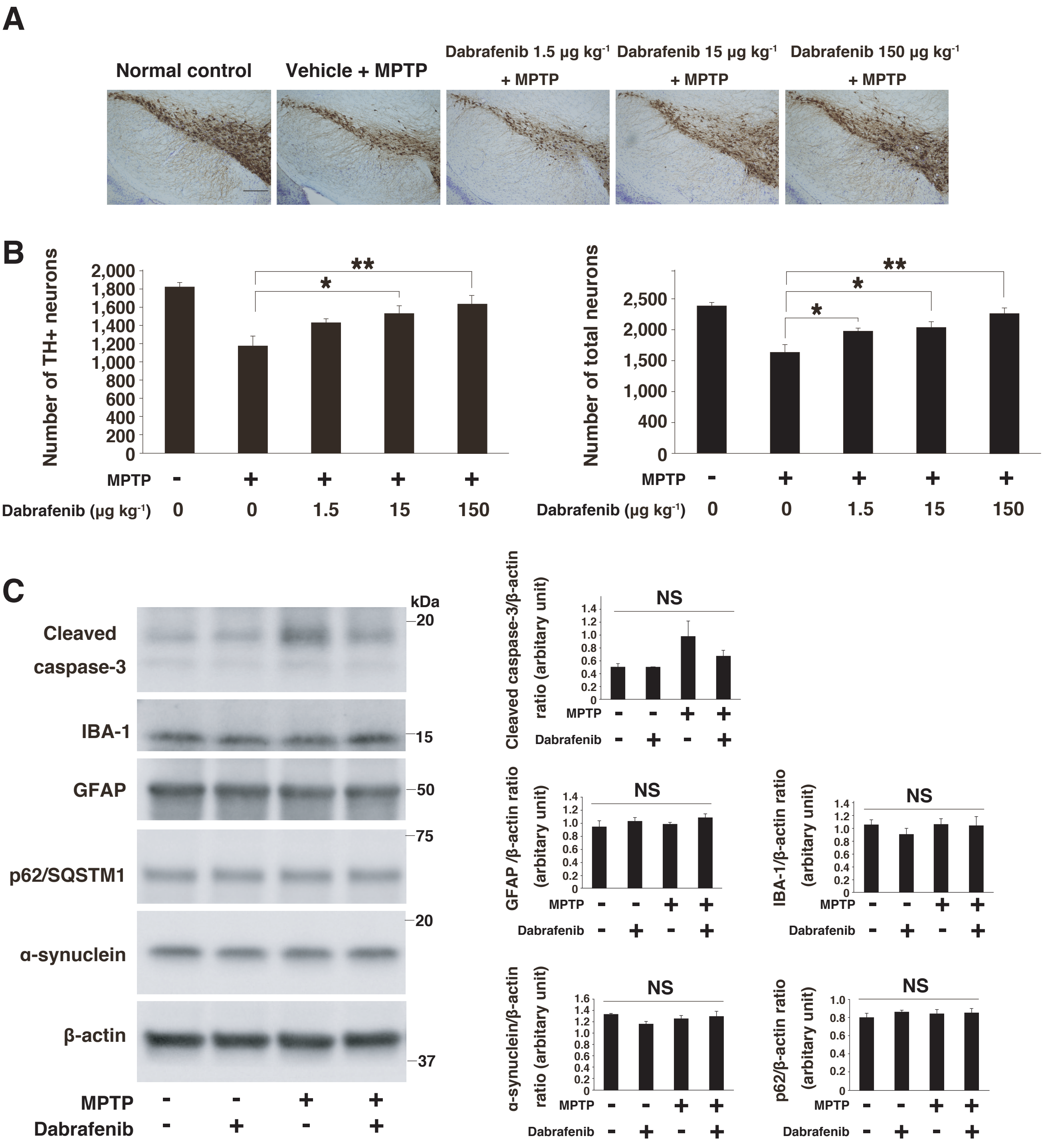

Supplementary Figure S3. Dabrafenib showed cytoprotective effects at one week after MPTP intoxication.

(A) Immunohistochemistry for TH on midbrain sections. Scale bar represents 200 μm.

(B) Stereological counts of TH-positive and total neurons in the substantia nigra pars compacta at one week after MPTP intoxication. Data represent mean ± s.e.m. *, $p < 0.05$ and **, $p < 0.01$ compared with vehicle + MPTP group (one-way ANOVA followed by the Tukey post-hoc test; n = 3-5 per group).
(C) Immunoblotting for cleaved caspase-3, α-synuclein, p62, GFAP, and IBA-1 at three weeks after MPTP intoxication. Data represent mean ± s.e.m. (one-way ANOVA followed by the Tukey post-hoc test; n = 3-4 per group).

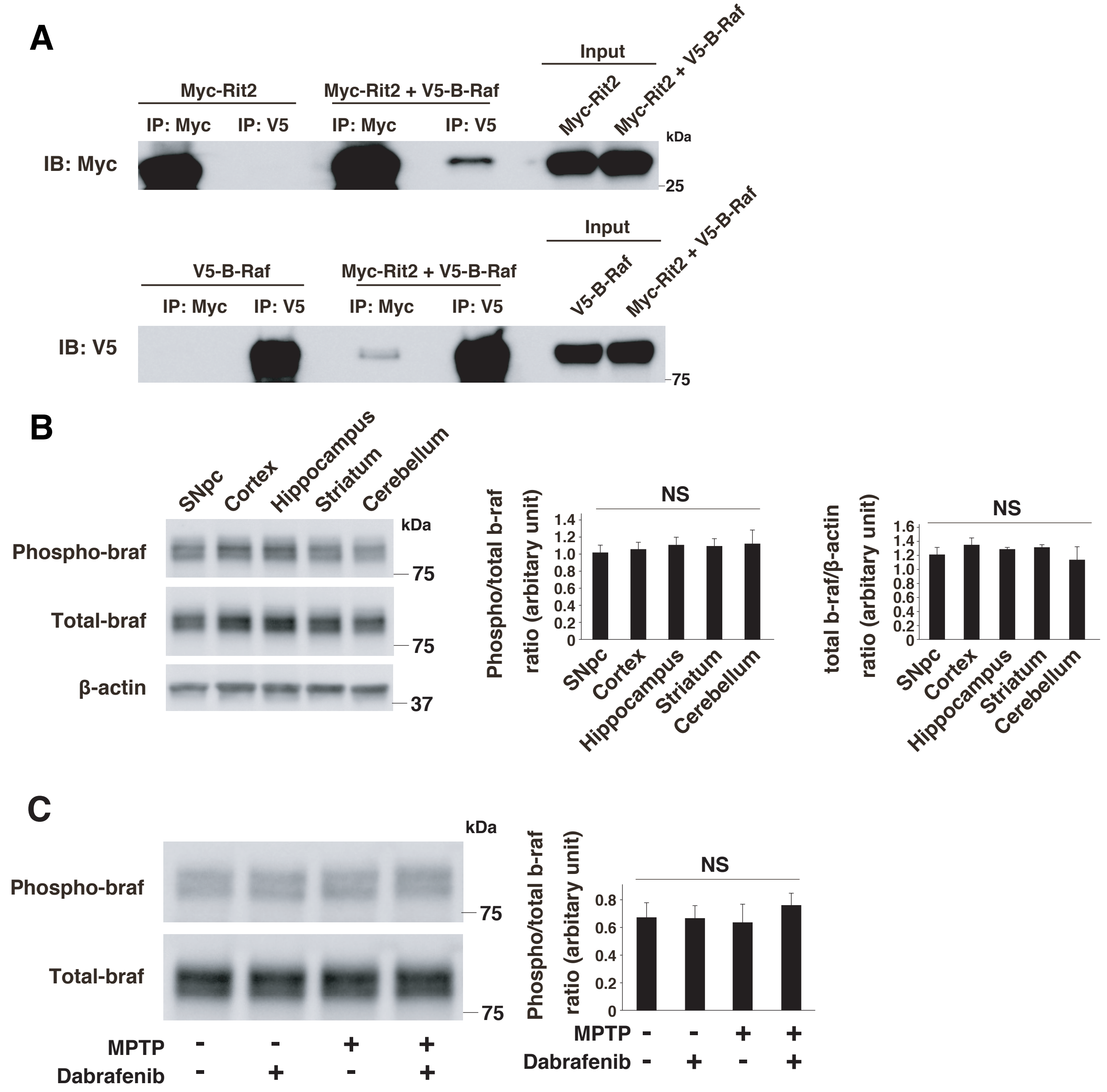

Supplementary Figure S4. Interaction between Rit2 and B-Raf.

(A) Co-immunoprecipitation of Rit2 and B-Raf expressed in HEK293T cells. In lysates of co-transfected cells, the precipitated Rit2 or B-Raf was detected by immunoblotting with an anti-c-Myc or anti-V5 antibody. The results are representative of three independent experiments.

(B) Western blotting for phospho- and total B-Raf in SNpc, the cortex, hippocampus, striatum, and cerebellum of control mice. Data represent mean ± s.e.m. (one-way ANOVA followed by the Tukey post-hoc test; n = 3-4 per group).

(C) Western blotting for phospho- and total B-Raf following exposure to MPTP and/or dabrafenib at three weeks after MPTP intoxication. Data represent mean ± s.e.m. (one-way ANOVA followed by the Tukey post-hoc test; n = 3-4 per group).

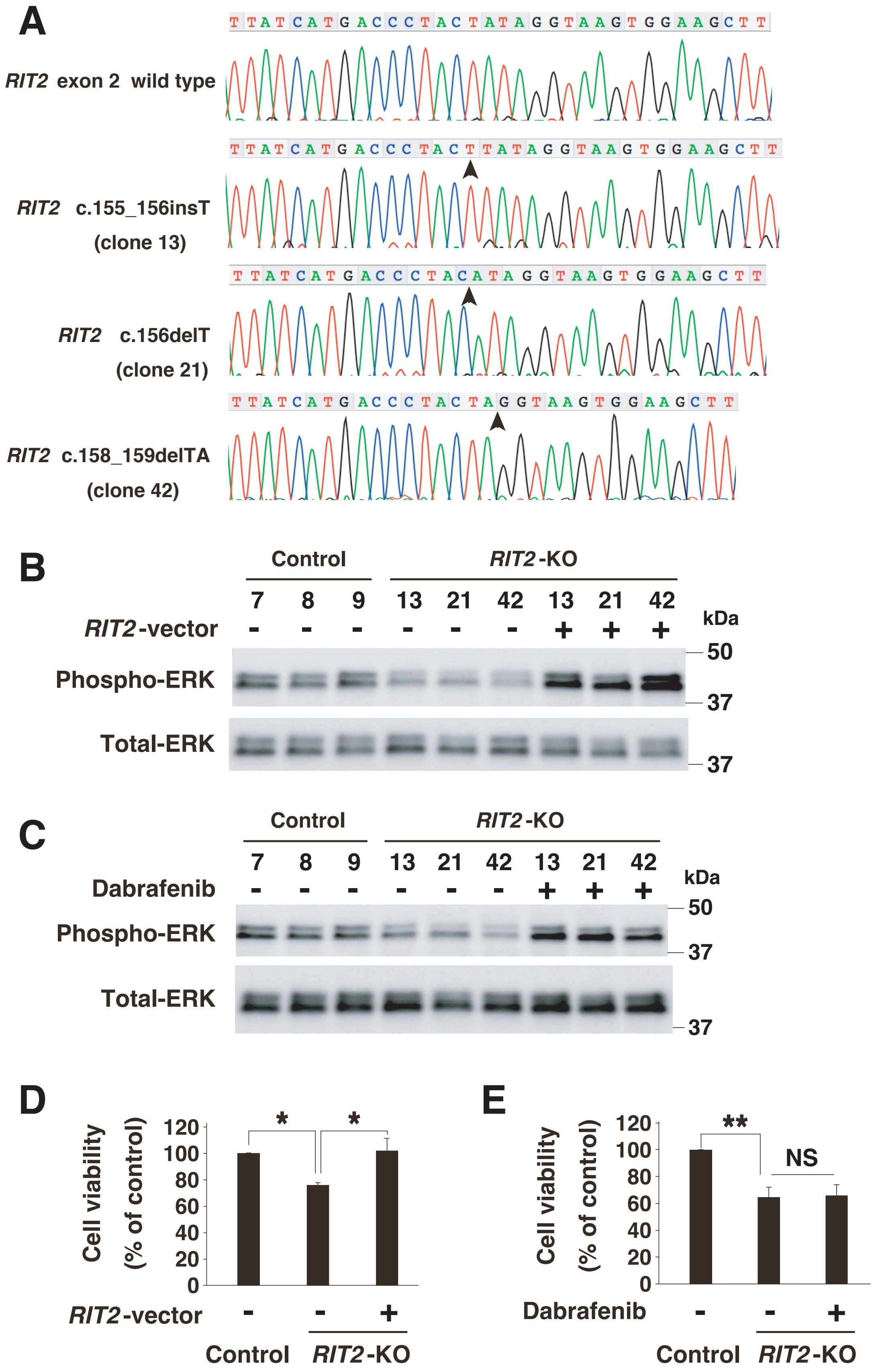

A
TTATCATGACCCTACTATAGGTAAGTGGAAGCTT
RIT2 exon 2 wild type
TTATCATGACCCTACTTATAGGTAAGTGGAAGCTT
RIT2 c.155_156insT
(clone 13)
TTATCATGACCCTACATAGGTAAGTGGAAGCTT
RIT2 c.156delT
(clone 21)
TTATCATGACCCTACTAGGTAAGTGGAAGCTT
RIT2 c.158_159delTA
(clone 42)
B
Control
RIT2-KO
7 8 9 13 21 42 13 21 42
RIT2-vector - - - - - - + + +
kDa
50
Phospho-ERK
37
Total-ERK
37
C
Control
RIT2-KO
7 8 9 13 21 42 13 21 42
Dabrafenib - - - - - - + + +
kDa
50
Phospho-ERK
37
Total-ERK
37
D
Cell viability (% of control)
120 100 80 60 40 20 0
* *
RIT2-vector - - +
Control RIT2-KO
E
Cell viability (% of control)
120 100 80 60 40 20 0
** NS
Dabrafenib - - +
Control RIT2-KO

Supplementary Figure S5. Confirmation of each *RIT2* gene mutation.

(A) CRISPR/Cas9-based frameshift mutations in exon 2 of the *RIT2* gene in SH-SY5Y cells were confirmed by sequencing. Black arrowheads indicate each insertion/deletion.

(B) Expression of phospho-ERK was reduced in *RIT2*-KO clones (13, 21, 42) compared with the control clones (7, 8, 9). Overexpression of *RIT2* in each *RIT2*-KO clone restored the phosphorylation of ERK.

(C) Dabrafenib upregulated the expression of phospho-ERK in each *RIT2*-KO clone.

(D) Cell viability in control and *RIT2*-KO cells. Cells were infected with a lentiviral vector overexpressing *RIT2* (MOI of 10), and cell viability was measured 48 hours after lentiviral transfection. Data represent the mean ± s.e.m. *, $p < 0.05$ compared with *RIT2*-KO cells treated with an empty vector (one-way ANOVA with the Tukey post-hoc test; n = 4 per group).

(E) *RIT2*-KO cells were treated with dabrafenib, and cell viability was measured after 48 hours of treatment. Data represent the mean ± s.e.m. **, $p < 0.01$ compared with *RIT2*-KO cells treated with DMSO; NS, not significant (one-way ANOVA with the Tukey post-hoc test; n = 4 per group).

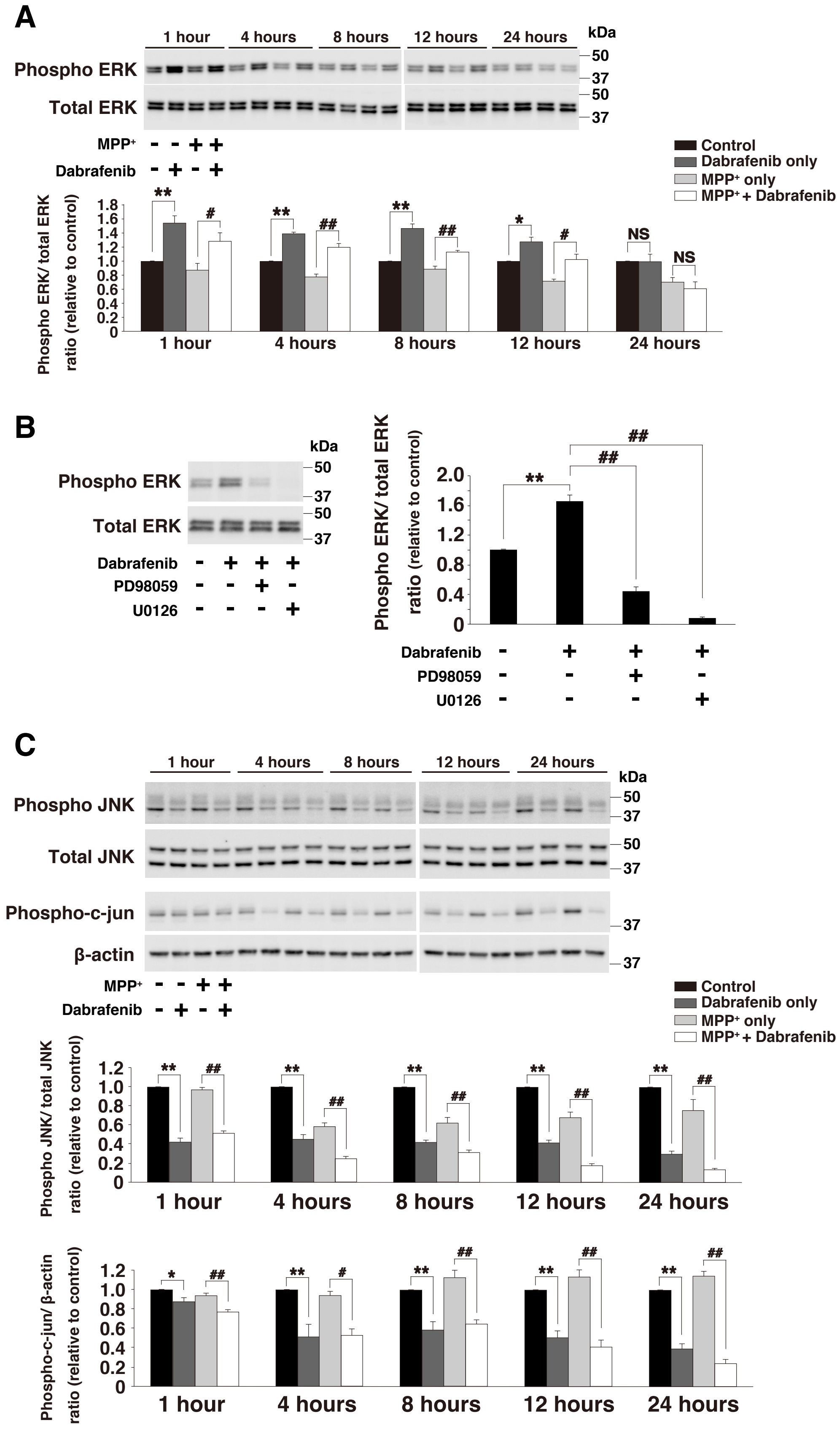

A
1 hour
4 hours
8 hours
12 hours
24 hours
kDa
Phospho ERK
Total ERK
50
37
MPP+
Dabrafenib
Control
Dabrafenib only
MPP+ only
MPP+ + Dabrafenib
Phospho ERK/ total ERK ratio (relative to control)
NS
B
Phospho ERK
Total ERK
Dabrafenib
PD98059
U0126
Phospho ERK/ total ERK ratio (relative to control)
C
Phospho JNK
Total JNK
Phospho-c-jun
β-actin
Phospho JNK/ total JNK ratio (relative to control)
Phospho-c-jun/ β-actin ratio (relative to control)

Supplementary Figure S6. Western blot analysis of the sequential changes in phosphorylated ERK, JNK, and c-jun expression levels after drug treatment.

(A) SH-SY5Y cells were exposed to dabrafenib (5 μM) and/or MPP^+ (3 mM) for various times up to 24 hours. Data represent the mean ± s.e.m. *, $p < 0.05$, and **, $p < 0.01$ compared with control; #, $p < 0.05$, and ##, $p < 0.01$ compared with MPP^+ alone (one-way ANOVA followed by the Tukey post-hoc test; n = 3 per group).

(B) The ERK inhibitors PD98059 (30 μM) and U0126 (3 μM) reduced the expression of phosphorylated ERK by dabrafenib (5 μM) in SH-SY5Y cells at one hour after drug treatment. Data represent the mean ± s.e.m. **, $p < 0.01$ compared with control; ##, $p < 0.01$ compared with dabrafenib alone (one-way ANOVA followed by the Tukey post-hoc test; n = 3 per group).

(C) SH-SY5Y cells were exposed to dabrafenib (5 μM) and/or MPP^+ (3 mM) for various periods of time up to 24 hours.

Data represent the mean ± s.e.m. *, $p < 0.05$ and **, $p < 0.01$ compared with control; ##, $p < 0.01$ compared with MPP^+ alone (one-way ANOVA followed by the Tukey post-hoc test; n = 3 per group).

Supplementary Table S1. Meta-analysis for rs4130047 by PDGene.

MyMeta result for rs4130047 To change your data for this meta-analysis please click here

Polymorphism	Location (hg19)	Gene	Ethnicity	# Samples	# Studies	Allele contrast	Meta OR (95%CI)	Meta P value	I2 (95%CI)
rs4130047	chr18:40678235	RIT2	All: C, J	NA	15	C vs. T	1.11 (1.08 - 1.15)	6.43e-11	24 (0 - 59)
rs4130047	chr18:40678235	RIT2	C	NA	14	C vs. T	1.1 (1.06 - 1.14)	4.52e-08	0 (0 - 53)

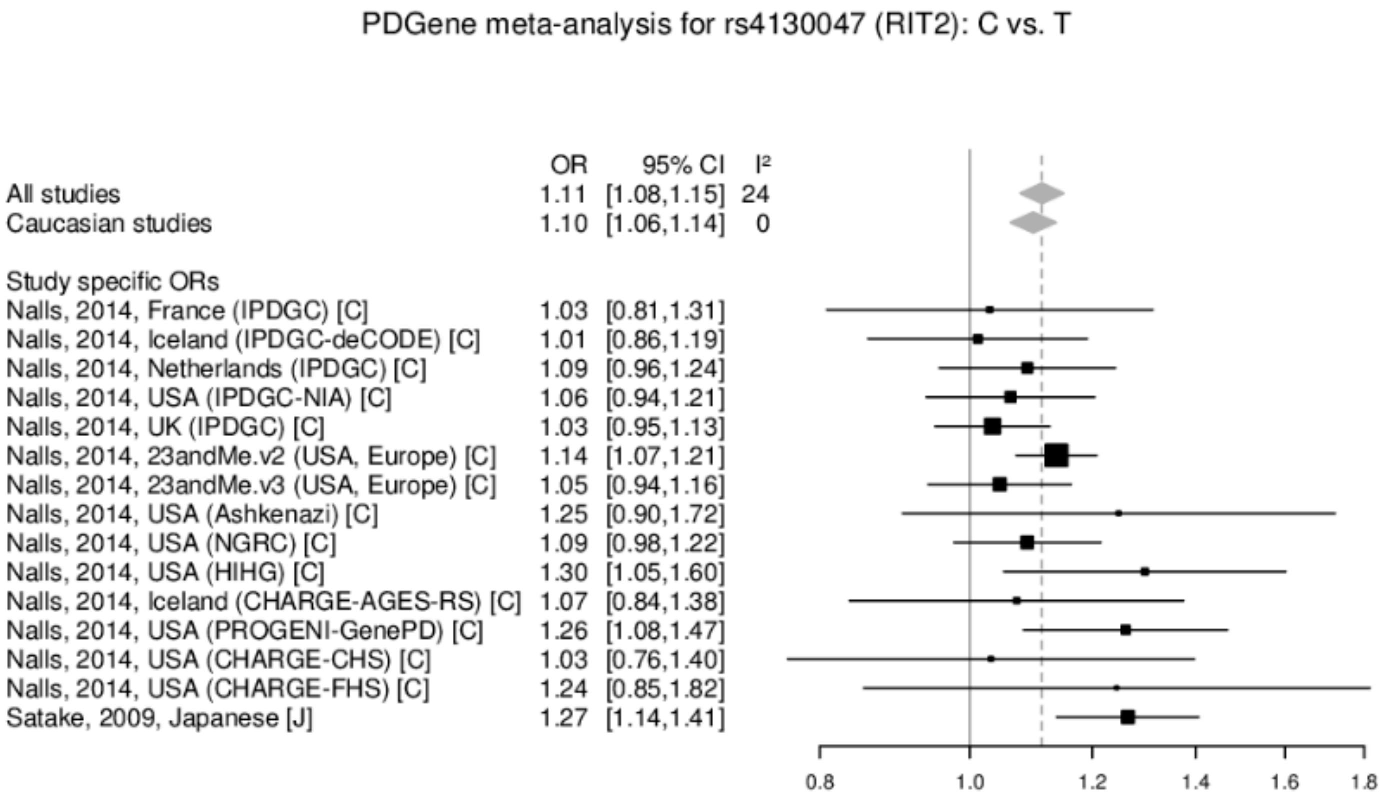

[C], Caucasian; [J], Japanese
